# Introducing a Rapid DNA Analysis Procedure for Crime Scene Samples Outside of the Laboratory—A Field Experiment

**DOI:** 10.3390/s23084153

**Published:** 2023-04-21

**Authors:** Rosanne de Roo, Anna Mapes, Merel van Cooten, Britt van Hooff, Sander Kneppers, Bas Kokshoorn, Thalassa Valkenburg, Christianne de Poot

**Affiliations:** 1 Forensic Science Department, Amsterdam University of Applied Sciences, Tafelbergweg 51, 1105 BD Amsterdam, The Netherlands; 2 Midden-Nederland Police Department, Forensic Investigative Division, 1276 KA Huizen, The Netherlands; 3 Amsterdam Police Department, Forensic Investigative Division, 1014 BA Amsterdam, The Netherlands; 4 Division Biological Traces, Netherlands Forensic Institute, 2497 GB The Hague, The Netherlands; 5 Police Academy, 7334 AC Apeldoorn, The Netherlands; 6 Department of Criminal Law and Criminology, Faculty of Law, VU University, 1081 HV Amsterdam, The Netherlands

**Keywords:** rapid DNA analysis, RapidHIT, forensic, criminal investigation

## Abstract

Technological innovations enable rapid DNA analysis implementation possibilities. Concordantly, rapid DNA devices are being used in practice. However, the effects of implementing rapid DNA technologies in the crime scene investigation procedure have only been evaluated to a limited extent. In this study a field experiment was set up comparing 47 real crime scene cases following a rapid DNA analysis procedure outside of the laboratory (decentral), with 50 cases following the regular DNA analysis procedure at the forensic laboratory. The impact on duration of the investigative process, and on the quality of the analyzed trace results (97 blood and 38 saliva traces) was measured. The results of the study show that the duration of the investigation process has been significantly reduced in cases where the decentral rapid DNA procedure was deployed, compared to cases where the regular procedure was used. Most of the delay in the regular process lies in the procedural steps during the police investigation, not in the DNA analysis, which highlights the importance of an effective work process and having sufficient capacity available. This study also shows that rapid DNA techniques are less sensitive than regular DNA analysis equipment. The device used in this study was only to a limited extent suitable for the analysis of saliva traces secured at the crime scene and can mainly be used for the analysis of visible blood traces with an expected high DNA quantity of a single donor.

## 1. Introduction

The use of DNA analysis in the process of criminal investigation and prosecution has grown exponentially over the past decades and is still increasing [1,2,3,4]. DNA analysis of biological traces and subsequent database searches can lead to investigative leads, the identification or exclusion of suspects, contribute to the reconstruction of an incident, or provide evidence against suspects [2,3,5]. During the crime scene investigation, items and trace evidence are secured at the scene. After selection, the most promising biological traces will be sent for DNA analysis. The resulting DNA profiles can be compared with the profiles of reference DNA profiles (e.g., potential suspects, victims within a case), and with a forensic DNA database (*In the Netherlands, this database contains profiles of suspects, convicts, trace material secured at crime scenes, and deceased victims of unsolved crimes*) for criminal cases. After this, the results are reported back to the investigation team.

Worldwide turnaround times for DNA results (time from DNA sampling at the crime scene or the (police) laboratory to DNA report) are longer than desired and can take weeks or months [1,6,7,8]. This asks for ‘rapid’ solutions, especially during the investigation phase, where forensic evidence increasingly influences the direction and, thereby, the effectiveness of the investigation [9].

Research has shown that a faster criminal investigation can contribute to a better crime approach (see among others [10,11]), and that swift action by the police can even double the number of solved cases [12]. Technology-driven innovations, such as “lab on a chip” and miniaturization of computers, radically changed forensic implementation possibilities [13]. Promising technologies became available, enabling rapid DNA analyses outside of the classical laboratory environment [14,15,16]. These innovations are in line with the great need articulated by the entire criminal justice chain (from crime scene to court) for rapid DNA results, and the desirability of laboratory analyses at the crime scene [17].

The current available rapid (mobile (*The ANDE is sold as being a mobile solution. The RapidHit and RapidHit ID are retailed as equipment for fixed locations (e.g., booking stations)*)) DNA technologies, the ANDE, Rapid ID, and RapidHIT, are less sensitive and robust than DNA analysis performed via the regular procedures in laboratories [18,19]. Rapid techniques are therefore prone to produce incomplete results when low DNA concentrations are present [15,20] and they are less suitable for analyzing complex mixture profiles [21,22,23], which is the trade-off for the advantages of speed and mobility.

Rapid DNA devices are successfully being used in practice for reference buccal samples and disaster victim identification samples, which are cell rich sources [16,24]. The techniques are also applied on less cell rich samples, such as actual crime scenes traces, of which the results vary [19,25,26,27]. For now, these technologies are presumed to be mainly suitable for crime scene traces with a high probability of obtaining a full DNA profile, namely blood and saliva traces presumably originating from a single donor [18,28].

The effects of implementing rapid DNA technologies in the crime scene investigation procedure have only been evaluated to a limited extent. The impact of implementing rapid technologies at the crime scene has been studied on mock crime scenes with fictive rapid analysis tools [1,29]. These studies show that the implementation of rapid identification techniques, such as tools for rapid analysis and comparison of DNA and fingermarks, can be efficient and effective in the investigative practice, both when it comes to a rapid identification of offenders [30] as well as the quality of the scenario reconstruction [31]. Next to this, the European Network of Forensic Science Institutes (ENSFI) and the Scientific Working Group on DNA Analysis Methods (SWGDAM) have set up additional requirements that should be taken into account before rapid (mobile) DNA technologies can be used for crime scene traces, emphasizing that the use of rapid technologies with crime scene traces should be handled with caution [32].

With the desire and available means for rapid DNA procedures, several large projects (Snelle-ID lijn, snelle DNA-straat, LocalDNA [33,34,35]) were initiated in the Netherlands, to investigate the effect of different (mobile) rapid procedures and gain more insight into the results, application possibilities, and impact of rapid information at the start of the investigative process, compared to the regular procedure. One of these projects is LocalDNA. In this project we set up a field experiment, comparing real crime cases and crime scene traces either following a rapid DNA procedure outside of the laboratory (decentral procedure), or a regular DNA analysis procedure at the forensic laboratory. These cases were followed from the start of the crime scene investigation until the apprehension of the suspect.

This study is one of the first studies investigating the impact of a rapid DNA procedure compared to a regular DNA procedure. More precisely, this is the first study that investigates the influence of the RapidHIT200 on blood and or saliva traces secured at a crime scene and the impact of these rapid DNA results on the investigative process.

## 2. Materials and Methods

### 2.1. Rapid DNA Device

In this study, we used the RapidHit R-DNA-DB08 direct PCR analysis device (*The RapidHit used in this study is now an obsolete device and is no longer manufactured (in this form) by Thermofisher Scientific. In this study, this device was used to test the principle of a decentralised procedure*) (Thermofisher Scientific, n.d., Waltham, MA, USA; Holland & Wendt, 2015) to perform the DNA analysis in the decentral rapid DNA procedure. This device is capable of analyzing 24 DNA markers and is suitable for analyzing 5 biological samples per run (*The cartridge of the equipment consists of 8 lanes with 3 lanes used for negative, positive and blank control samples and 5 lanes left for samples*). The device is fully automatic and able to obtain raw DNA data from samples within 2 to 3 h that can be processed, interpreted, and compared to the DNA database. The RapidHit was purchased by the National Criminal Investigation Service (*Dienst Landelijke Recherche*) of the Dutch National Police and is located in a vehicle, making it possible to use it in a mobile or decentral (outside of the laboratory) setting. The decentralized process of rapid DNA analysis is validated and accredited for blood and saliva samples [36].

### 2.2. Design

This study aimed to monitor 50 cases following the decentral rapid DNA procedure and, in parallel, 50 similar cases following the regular DNA procedure. The selected cases were analyzed with the aid of an extensive analysis model consisting of over 800 variables covering general case information, the timeline, enrolled capacity, quality of the investigation and the traces, and detectives’ experience with the different DNA procedures. Variables on the duration and quality of the investigative process were analyzed to investigate the impact of the two procedures on the criminal investigation process. In this paper, we focus on the duration of the criminal investigation process and the quality of the DNA analysis results.

**Figure 1:**
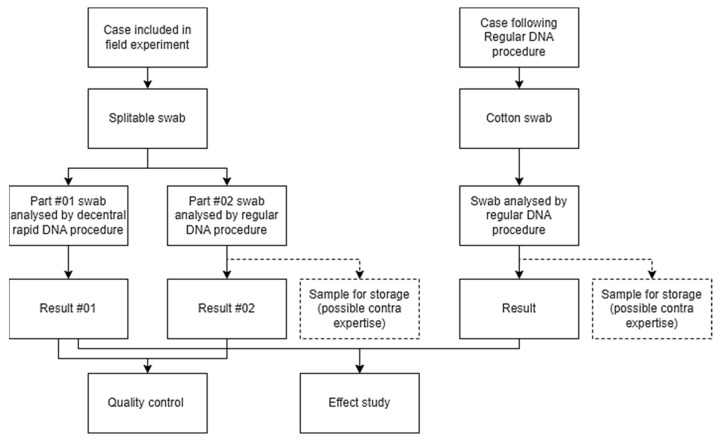
Overview of research design.

To compare results obtained with a decentral rapid DNA analysis procedure and the traditional procedure (quality control), all DNA traces in the field experiment were sampled with a splitable swab (*Copan’s splitable 4N6 FLOQ Swabs Genetics was validated for DNA profiling using the RapidHit and by regular DNA profiling at the NFI* [37]). This splitable swab ensures that the trace material is sampled once and then split: one half of the swab was analyzed with the rapid DNA technology and the second half of the swab followed the regular DNA procedure at the Netherlands Forensic Institute (NFI). Forensic investigators were trained to sample with a rotary motion, in an attempt to obtain a homogeneous distribution of the trace on the swab. The swab was split in a controlled environment by a trained lab technician. A schematic overview of the design of the study is shown in Figure 1.

### 2.3. Inclusion Criteria Cases and Traces

In the period of November 2020–July 2021, both serious crimes (e.g., homicides, robberies, violent crimes) and volume crimes (e.g., property crime, vandalism) committed in the two participating police regions (Police region *Amsterdam* and police region *Midden-Nederland*) were eligible for this field experiment. Inclusion criteria encompassed the following: (1) the crime scene investigation was conducted by a forensic investigator, (2) assumed blood and/or saliva traces presumably from one donor were present at the scene, and (3) a public prosecutor had given permission (*In the Netherlands, the (forensic) prosecutor is formally in charge of conducting the investigation and formally orders (follow-up) investigations*). For practical reasons, the field experiment was (mainly) deployed on weekdays during office hours.

In the period of December 2018–November 2019, in total 50 serious and volume crime cases that followed the regular DNA analysis procedure but would have met the deployment criteria for the decentral rapid procedure were selected and analyzed retrospectively. To determine whether a case would have qualified for the decentral rapid DNA procedure, interviews were conducted with the forensic investigators and the investigation leader. Those cases that met the criteria and would have been selected for the decentral rapid DNA procedure were included in the study as a comparison group.

#### 2.3.1. Exception Trace Sampling

Blood and saliva traces were sampled with the previously described splitable swab method. In the case of cigarette butts (saliva traces) we used another procedure. In regular DNA testing at the NFI, (part of) the cigarette butt is examined. Cigarette butts contain substances derived from tobacco and its burning; therefore, purification steps are taken to remove these substances. In the direct PCR analysis with the RapidHit, no purification steps are performed; therefore, this technique is not suitable for examining cigarette butts (and other samples with similar inhibitory substances). Consequently, cigarette butts were not directly examined but swabbed with a regular cotton swab which was analyzed in the RapidHit, thereby attempting to reduce the amount of inhibitors in the sample. For analysis in the regular procedure the cigarette butt was sent to the laboratory, where part of the paper wrapping of the filter of the cigarette was sampled and analyzed as a control, rather than half of a splitable swab as with the other samples.

#### 2.3.2. Trace Result Categorization

To analyze and compare the trace results of the decentral rapid DNA procedure and the regular procedure, the trace results are divided into 4 categories: (1) ‘good DNA profile’: a DNA profile suitable for admission to the DNA database [38]; (2) ‘profile suitable for one-time comparison’: a DNA profile too complex for admission to the DNA database but suitable for comparison against database reference profiles through SmartRank [39]; (3) ‘profile suitable for comparison within a case’: a complex DNA profile but informative enough to compare to reference profiles within a case; (4) ‘no profile/unsuitable for comparison’. Note that, based on the validation of the system, DNA profiles obtained through the RapidHit analyses needed to be single source to be considered for comparison. Mixed DNA profiles were considered to be unsuitable for comparison. Mixed DNA profiles were considered for comparison in the regular procedure.

### 2.4. Procedures

For this study a new decentral rapid DNA working process was set up and evaluated during the study. Cases following the decentral rapid DNA procedure followed the steps listed below. For the regular DNA procedure, a simplified version of the procedural steps is also outlined below.

#### 2.4.1. Decentral Rapid DNA Procedure

Forensic investigators arrive at a crime scene at which potentially suitable traces (blood and/or saliva) for the decentral rapid DNA procedure are present. If such traces are found, the field experiment-coordinator is notified. Collectively, the suitability of the traces is determined based on the criteria: blood and/or saliva traces with a high probability of obtaining a full DNA profile from one donor. Permission for the decentral rapid DNA procedure is requested from the prosecutor, based on the case and proposed selection of traces. Upon agreement, the field experiment-coordinator notifies the forensic lab technicians of the police region concerned, the deployment coordinator of the rapid DNA device of the national police unit, and a DNA expert of the NFI. The rapid DNA device is moved to a suitable location (e.g., investigation site or police station).

The forensic investigator samples suitable traces with the splitable swab and photographs the samples and traces. The samples are handed over to the lab technicians of the relevant unit, who split the swab and hand over one half of each sample to the lab technician of the national unit, who enters the sample into the rapid device and starts the run (*Due to the accreditation criteria, only lab technicians of the national police unit were allowed to enter samples in the RapidHit device*). The second half of the swab is analyzed at a later time, following the regular procedure (see next paragraph). Upon completion of the analysis, the data generated by the RapidHit is transmitted via a secure data-connection to the NFI. At the NFI, analysts that are trained at analyzing the RapidHit system electropherogram data analyze the obtained DNA data. The resulting DNA profiles are interpreted by a DNA expert who also performs the comparison of the profiles (if applicable) with profiles within the case. The expert also initiates a DNA database search when the profiles meet the database search criteria. The results are first reported by telephone to the field experiment-coordinator, who informs the forensic investigators and forensic prosecutor. Within 24 h an official DNA expert report is sent by e-mail to the forensic prosecutor, field experiment-coordinator, and other usual recipients of DNA reports.

#### 2.4.2. Regular DNA Procedure (Simplified)

Forensic investigators arrive at a crime scene. The forensic investigator samples and photographs the samples and traces. If necessary, DNA sampling/pre-examination is carried out in the police laboratory by lab technicians. The collected samples are prioritized based on potential success rates and crime relatedness. Permission for DNA analysis is requested from the forensic prosecutor based on the case and proposed selection of traces. The samples are sent to a forensic laboratory, where the DNA samples are isolated, quantified, and amplified. Material (DNA extract) is separated and stored for possible future contra-analysis. The DNA experts analyze the obtained samples, interpret the DNA profiles, and compare the profiles (if applicable) with profiles within the case and with profiles stored in the DNA database for criminal cases. The results are reported in an official DNA expert report by e-mail to the forensic prosecutor and other usual recipients of DNA reports.

## 3. Results

Fifty cases that followed the regular procedure, encompassing 37 serious crime cases and 13 volume crime cases, and 47 cases (*The goal was to investigate 50 cases. During the study period, there was limited availability of the mobile DNA device. As a result, only 47 cases were investigated*) that followed the decentral rapid procedure, encompassing 16 serious crime cases and 31 volume crime cases were monitored. The impact of the used procedure on the investigative process is divided into two sections: (1) impact on duration of the investigative process and (2) impact on the quality of the trace results.

### 3.1. Duration of the Investigative Process

#### 3.1.1. Turnaround Times Decentral Rapid DNA Procedure

The turnaround time from the notification of a crime until DNA results were reported to all parties of the case in the decentral rapid DNA procedure (n = 47) averaged 46 h. The average time between reporting the crime and investigating the crime scene was 5.5 h. The crime scene investigation took an average of 1 h. The time between the start of the crime scene investigation and requesting the rapid DNA procedure averaged 7.5 h. The data suggests that these 7.5 h of ‘time loss’ were mainly related to the moment a crime is reported to the police. Many crimes take place in the evenings and on weekends. For this field experiment, the rapid DNA technology was (mostly) deployable on weekdays between 8 a.m. and 5 p.m. As a result, a relatively large amount of time was lost, not only between the crime scene investigation and requesting the RapidHit, but especially in the period between the implementation of the rapid DNA technology and the start of this DNA analysis on location, which averaged 28 h. Only in 26% (12 out of 47) of the cases could the rapid DNA procedure start on the same day as the crime scene investigation. In 36% of the cases (17 out of 47) the procedure was performed 1 day after the incident and in 38% of the cases (18 out of 47) the rapid DNA analysis was performed 2 or more days after the crime scene investigation. Generating the DNA profiles with the RapidHIT took an average of 2 to 2.5 h, after which the NFI communicated the results back within, on average, 1.5 h to the investigation leaders. Communicating the results between the forensic prosecutor, crime scene investigators, and apprehension team took an average of 2 h. Notable here is that communication with the forensic prosecutor and crime scene investigators was relatively quick (respectively, after an average of 20 and 35 min) however results were communicated to the teams responsible for the apprehension after an average 3 h. This average time is slightly higher as in some cases the results were not communicated to the apprehension team until several days after the reporting.

#### 3.1.2. Duration Investigative Process Decentral Rapid DNA Procedure vs. Regular Procedure

In order to understand the potential impact of the decentralized rapid DNA procedure on the duration of the investigation process and on the identification of suspects (*Throughout this paper we refer to ‘identification of suspect’ to summarize the results of DNA comparison (through the DNA database or with reference profiles in a case) and likelihood ratio calculations supporting the presence of DNA of an individual in the sample. Whether or not this resulted identification of the person of interest, and this person being considered a suspect are legal matters that are outside of the scope of this paper*) in comparison to the regular DNA procedure, all cases where an identification occurred as a result of a comparison with the DNA database and forensic investigators had a leading role in the identification of a suspects (regular DNA procedure, 11 of the 37 cases; rapid DNA procedure, 19 of 36 cases (*In 22 cases a donor was identified through a search in the DNA database. In 3/22 cases the apprehension team identified the suspect prior to the DNA database result. Therefore these 3 cases were excluded for this analysis.)*) were analyzed. In these cases, the rapid DNA technology can provide a potential acceleration in the investigative procedure. The date and time of different stages in the investigative procedure were recorded and used for this analysis, namely: report of the crime, start of the crime scene investigation, RapidHIT deployment, prioritization of traces, sending traces/data to the NFI, DNA report, and identification and apprehension or signaling of the suspect.

**Figure 2:**
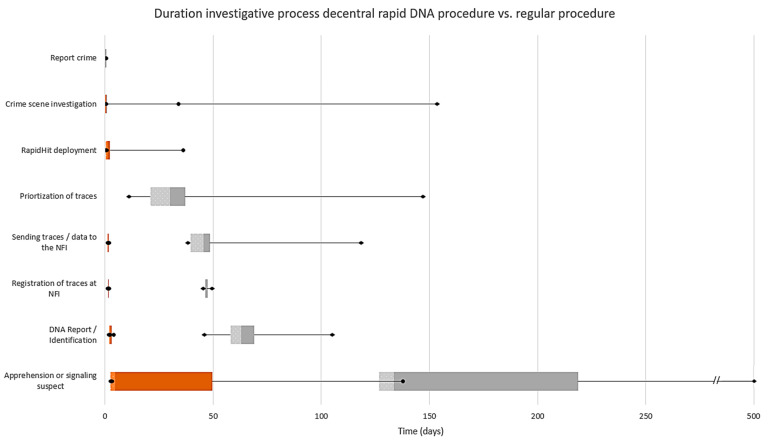
Horizontal box plots with the medians and quartiles categorized by the different stages of the investigative process over time (days). The orange plots show the decentral rapid DNA procedure. The grey plots show the regular DNA procedure.

As mentioned previously, the average time to identify a person via the decentral rapid DNA procedure (from the start of the crime scene investigation to the identification of the person as a result of a DNA database match) averaged 46 h ≈ 2 days (n = 19). Duration between the date of identification and the apprehension or signaling of a suspect averaged 20 days (median 4 days) in the decentral rapid procedure. In five out of the nineteen cases (26%), the suspect was apprehended within two days after the identification. In the regular procedure (n = 11), the time (in days) to identify a person (from the start of the crime scene investigation to identification) averaged 66 days (median 49 days). After crime scene investigation, it took on average 29 days (median 29 days) before traces were selected and prioritized by the police, after which it took on average another 16 days (median 4 days) before the traces were sent to the laboratory for DNA analysis. After arrival, traces were booked in (average 2 days; median 1 day), interpreted, and reported back within an average of 19 days (median 15 days). The average time between the date of identification and the apprehension or signaling of a suspect averaged 126 days (median 73 days) in cases following the regular procedure (n = 11). A more detailed timeline with the median and quartiles can be found in Figure 2.

There is a significant acceleration in the investigative process from the report of a crime until the apprehension or signaling of a suspect in the decentral rapid DNA procedure compared to the regular procedure (t(28) = 3.750, *p* = 0.001). An in-depth analysis of the data shows that there was a significant acceleration between the two procedures in the following steps of the process: ‘sending traces/data to the NFI‘ (t(28) = 3.181, *p* = 0.004); ‘DNA report/identification’ (t(28) = 2.275, *p* = 0.032) and ‘apprehension or signaling suspect‘ (t(28) = 5.609, *p* < 0.001). No significant acceleration between the two groups was seen in the duration of the ‘crime scene investigation’ (t(28) = 1.514, *p* = 0.159) and the ‘registration of traces at NFI’ (t(28) = 1.092, *p* = 0.284).

### 3.2. Quality of the Trace Results

In the 47 cases where the RapidHit was deployed, a total of 97 blood and 38 saliva traces were sampled with the splitable swab. Blood and saliva traces analyzed with the decentral rapid DNA procedure provided (non-complex) single DNA profiles in 65% of the blood traces (63 out of 97) and in 26% of saliva traces (10/38).

#### 3.2.1. Identifications

The trace results yielded at least one usable DNA profile in 37 of the 47 cases (79%) analyzed with the decentral rapid DNA procedure, which led to the identification of a potential donor of the trace in 25 of the 47 cases (*Previously, in section ‘Duration investigative process decentral rapid DNA procedure* vs. *regular procedure,’ 19 identifications were discussed with the decentral rapid DNA procedure in which forensic investigators were leading in identifying suspects. Here, all identifications obtained with the RapidHit are discussed*) (53%). In 22/25 cases, a potential donor was identified through a search in the national DNA database for criminal cases. In 19/22 cases, this was a blood trace and in the remaining 3 cases a saliva trace. In 3/25 cases, a match with a trace of known origin, a saliva reference sample, was found. Of the 25 identifications, 28% (7/25) were found in serious crime cases (5 through the DNA database, 2 through a ‘reference’) and 72% (18/25) were found in volume crime cases (17 through the DNA database and 1 through a ‘reference’).

The same traces analyzed with the regular procedure (second half of the splitable swab) provided (non-complex) single DNA profiles in 92% (89/97) of the blood traces and in 68% (26/38) of the saliva traces, giving at least one usable DNA profile in 45 of the 47 cases (96%), leading to an additional 19% identifications (9 of 47 cases; 4 blood traces, 5 saliva traces). Next to this, there was one case where the rapid procedure did yield a DNA profile suitable for comparison that could be compared manually once with individuals in the DNA database, but this comparison did not yield a match. The (more sensitive) laboratory analysis of this trace, on the other hand, did yield an identification with the DNA database. Subsequent analysis showed that rapid DNA analysis had generated a profile with only a few markers causing a DNA database identification with a low probative value, which did not result in an identification.

#### 3.2.2. Quality of the Generated DNA Profiles

With the rapid procedure, a good DNA profile (suitable for admission to the DNA database) was generated for 45% of the blood traces (44/97), while the regular procedure resulted in a good profile in 95% (92 of 97) of the blood traces. Saliva traces (n = 38) were divided into three subcategories: saliva, cigarette, and reference buccal swab samples. For saliva traces, a good DNA profile was produced for 8% (2/25) of the traces with the rapid procedure versus 56% (14/25) with the regular procedure. Swabs from seven cigarette butts were examined with the rapid procedure, none of which resulted in a good DNA profile (*During the field experiment, based on experience, stricter criteria for selecting saliva traces than for blood traces were implemented. Cases with a single cigarette butt (saliva trace) were no longer eligible for analysis with the rapid DNA equipment, and samples from face masks also proved unsuitable for deployment of the DNA analysis equipment*). The regular DNA analysis consisted of analyzing the cigarette butt’s filter paper in the extraction and resulted in a good profile for four butts. Four of the six reference buccal swabs resulted in good DNA profiles in the decentral rapid procedure vs. five in the regular procedure.

**Figure 3:**
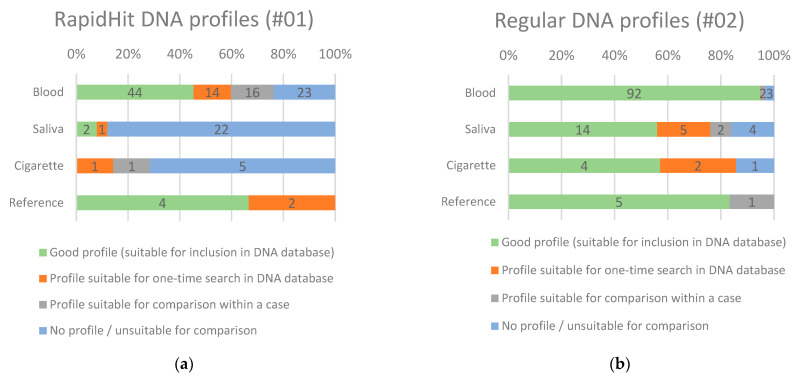
(**a**) DNA profiles generated by decentral rapid procedure. (**b**) DNA profiles generated by regular procedure.

In Figure 3, the results of the DNA profiles obtained with the decentral rapid DNA procedure are visualized and compared with the DNA profiles obtained by the regular procedure from the same samples. The percentage of a ‘good profile’ was significantly (*p* < 0.01) higher using the regular DNA examination compared to the rapid procedure for all types of traces—except the reference buccal swabs.

Analysis of the DNA markers showed that the quality of DNA profiles obtained with the rapid procedure is structurally lower compared to DNA profiles obtained by regular procedure; predominantly lower peak heights (low template DNA profiles) are observed for 43% of the traces analyzed with the rapid procedure (58/135) versus 11% in the regular procedure (15/135). For 62% of the traces analyzed with the rapid procedure (83/135), imbalance between the peaks and stochastic effects, such as allele and locus drop-out, occurred versus 17% in the regular procedure (23/135). Also, artifacts are often visible in the profiles obtained with the rapid procedure such as broadly spaced peaks, asymmetric peaks, signal pull-up and distorted baselines.

#### 3.2.3. Sensitivity RapidHit

The sensitivity of the RapidHit to derive a full DNA profile is set on the threshold of 0.25 μL of blood on a cotton swab by the company ThermoFisher [40]. Blood contains 0.020–0.040 μg DNA/μL blood [41,42], equaling that the stated threshold of 0.25 μL blood contains 5–10 ng DNA.

The RapidHit does not measure DNA quantity, therefore the DNA quantity of the analyzed samples by the rapid procedure is unknown. However, the amount of DNA in the laboratory samples was quantified in the regular procedure. Evidently, the quantities on both parts of the splitable swab cannot be assumed to be exactly the same. Yet, due to the used swabbing technique it can be assumed that the quantities are comparable to some extent. For this analysis it is assumed that the amount of DNA in the swab half analyzed with the RapidHit is equal to the amount of DNA in the other half analyzed in the laboratory.

**Figure A1:**
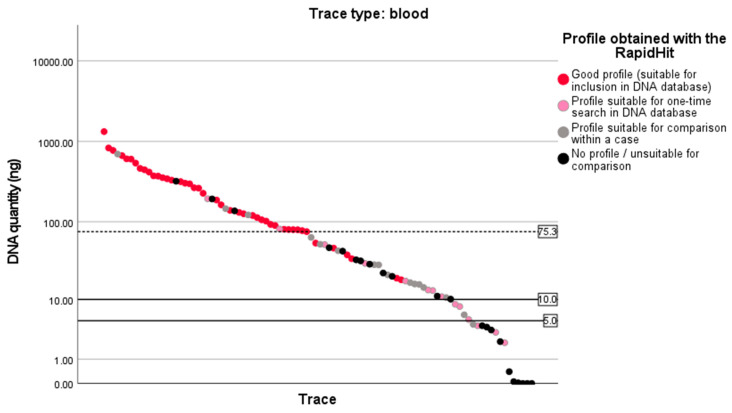
The distribution of the DNA quantity of blood traces (n = 97) of the laboratory samples analyzed by the regular procedure linked with the profiles generated by the RapidHit. The dotted line is the point from which mainly (86%) ‘good profiles’ were produced. The solid lines are the stated threshold of the RapidHit to derive a full DNA profile 0.25 μL of blood, containing 5–10 ng of DNA.

**Figure A2:**
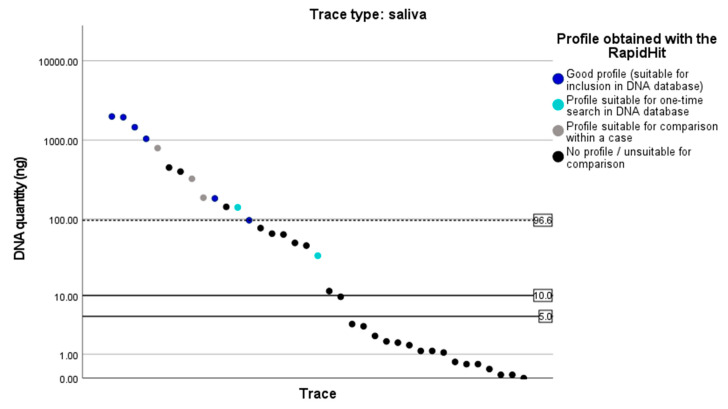
The distribution of the DNA quantity of saliva traces (n = 38) of the laboratory samples analyzed by the regular procedure linked with the profiles generated by the RapidHit. The dotted line is the point from which 46% of ‘good profiles’ were obtained. The solid lines are the stated threshold of the RapidHit to derive a full DNA profile 0.25 μL of blood, containing 5–10 ng.

Starting at a DNA quantity of 75.3 ng DNA for blood traces, mainly ‘good DNA profiles’ were observed in 87% of the blood samples. For saliva traces, 46% of saliva samples resulted in a ‘good DNA profile’ when the sample contained at least 96.6 ng of DNA. With lower quantities, usable DNA profiles were obtained sporadically. The lowest amount of DNA from which the RapidHit could extract a DNA profile usable for comparison with the DNA database was 2.2 ng DNA for blood traces and 33.9 ng DNA for saliva traces. The distribution of the DNA quantity of blood (n = 97) and saliva traces (n = 38) of the laboratory results linked with the profiles generated by the RapidHit is shown in Figure A1 and Figure A2 in the Appendix A.

Based on ThermoFisher’s stated threshold of being able to derive a full DNA profile with 5–10 ng DNA (0.25 μL of applied blood on a swab), we would have expected that the DNA samples in our study would have yielded a ‘good’ DNA profile more often.

## 4. Discussion

In this study, the effect of a decentralized (outside the laboratory) rapid DNA technique was investigated by comparing 47 real criminal cases where 135 real crime scene samples were analyzed with this procedure to 50 cases following the regular DNA procedure.

### 4.1. Duration Investigative Process

The decentral rapid DNA procedure has strongly accelerated the duration of the investigative process compared to the regular investigative procedure (average 22 days vs. 192 days) in cases where a person was identified as a result of identification with the DNA database. This result is mainly achieved by: (1) the acceleration on procedural steps before traces can be send to the laboratory in the regular procedure (average 45 days) vs. deployment of the RapidHit (average 2 days); (2) the acceleration of the analysis (average 2–2.5 h) and interpretation (average 1.5 h) of the DNA results in the decentral rapid DNA procedure vs. DNA analysis of traces in the regular procedure (average 19 days); (3) and apprehension or signaling of a suspect averaged 20 days in the decentral rapid DNA procedure vs. 126 days in cases following the regular procedure.

There may be a variety of reasons why suspects are not apprehended immediately upon identification. For example, in one case following the decentral rapid DNA procedure, it was decided to wait 6 days before apprehending a suspect because more evidence needed to be collected. In another case, a DNA profile in the DNA database needed to be ‘upgraded’ to ensure the reliability of the identification before the suspect could be apprehended, spanning 28 days. In a third case, it took a relatively long time before a suspect could be signaled as the cases concerned a Prüm (*An international comparison of DNA profiles between (EU) member countries. The comparison is done in writing through mutual legal assistance requests or automated based on EU legislation based on the Prüm treaty*) identification and the paperwork took 131 days. There were also instances when a suspect could not be apprehended immediately due to lack of capacity i.e., available police.

In general, the time of the whole investigative process seems to have been significantly reduced compared to the regular procedure. However, the low number of cases and variability between the cases asks for cautious statements about the added value of the procedure for the processing time from identification to apprehension. This is partly because in the decentral rapid DNA procedure the field experiment leaders, crime scene investigators, forensic prosecutor, and case officers proactively shared identifying information, which affects the speed of the apprehension or signaling of a suspect. Implicitly and explicitly, there has been prioritization to the cases following the rapid procedure.

### 4.2. Trace Results

In total, in 53% of the cases (25/47 cases) a match with a donor was found using the RapidHit. A fast identification was obtained in 40% of the cases (19/47 cases) through a blood trace, and in 13% of the cases through a saliva trace (6/47). Of these 25 identifications, 28% (7/25) were found in serious crime cases and 72% (18/25) in volume crime cases. This indicates that the decentralized rapid DNA procedure is more suitable for volume crime cases with a relatively high percentage of repeat offenders, whose DNA profiles are relatively often included in the DNA database.

Due to the lower sensitivity of the RapidHit compared to the DNA analysis in the regular procedure, identifying information was lost in 19% of the cases following the rapid procedure, where analysis at the laboratory did yield an identification (this was the case with 4 blood traces and 5 saliva traces). The RapidHit generated a good DNA profile (suitable for admission to the DNA database) in 42% of the blood traces (41/97); while the regular procedure resulted in a good profile in 94% (91 of 97) of these traces. Although these are apparently reasonable results, blood traces do sacrifice a great deal in terms of the quality of the DNA profile in the rapid DNA analysis procedure. For saliva traces a good DNA profile was produced for 8% (2/25) of the traces with the rapid procedure versus 52% (13/25) with the regular procedure. By default saliva contains less DNA than blood, possibly explaining the lower results (*Saliva is also a more complex matrix intended for degradation of biological material, which can also explain the lower DNA results*). Next to this, the uncertainty in collecting invisible saliva traces based on contextual information compared to directly sampling visible blood stains could also be a reason for the reduced number of useful DNA profiles. The results of regular DNA analysis of these saliva traces indicated that many of them are not promising traces, arguing that only large/visible saliva traces with a high success rate should be selected for analysis with the RapidHit. None of the saliva traces sampled from the cigarette butts examined with the rapid DNA analysis procedure resulted in a good DNA profile, demonstrating that this technique is unsuitable for examining sampled cigarette butts. These limited DNA results for blood and saliva traces were expected and confirm the results of the validation study performed by the NFI [37]. The results also emphasize that the use of the RapidHit 200 for analyzing crime scene traces should be handled with caution and additional requirements as stated by the ENSFI, SWGDAM and Rapid DNA task group, should be taken into account before rapid (mobile) DNA technologies can be used for crime scene traces [32].

#### 4.2.1. Contamination

In one case a noteworthy result was found. One swab derived from a cigarette butt was examined using the rapid procedure. This sample was examined in lane 1 of the cartridge. Lanes 2 through 5 were left empty. When analyzing the data, it was found that lane 1 did not produce a DNA profile. However, in lane 2, a single, almost complete DNA profile of an unknown man was obtained. To find the cause of this possible contamination, the cigarette butt sample from lane 1 was subsequently submitted for regular DNA testing at the NFI. This resulted in a single DNA profile, that did not match the DNA profile of the unknown man from lane 2. The DNA profile of the unknown man from lane 2 was compared with the national elimination DNA database and once with the Dutch DNA database for criminal cases. The profile was also compared with employees of the manufacturer of the cartridge. None of the comparisons resulted in a match. This raises the question how error- and contamination-prone and usable this equipment is for crime scene traces, which may warrant additional research in this area.

#### 4.2.2. Mistyping

For six DNA profiles obtained with the rapid procedure, one or two STR markers were mistyped based on the control analysis performed. All six profiles were low-template DNA profiles, for which this is a well-known phenomenon. Mistyping can lead to a loss of probative value of possible matches or to differences in the list of possible matches after searching a DNA database. No incorrect identifications occurred due to mistyping in the field experiment cases. Yet, additional research should indicate the probability of incorrect individualization.

#### 4.2.3. Multiple Donors

In four samples (two blood and two saliva), the rapid procedure resulted in a DNA profile with characteristics of one person whereas the quality control resulted in a DNA mixture profile of two or more persons for these samples. This is caused by the difference in sensitivity between the two techniques. The main donor of the four samples was found by the rapid procedure, but the additional donors who contributed relatively little DNA, were not detected. It can be very relevant in a case to know whether DNA from one individual is present or DNA from more individuals is present in a sample. For example, in addition to the main profile of the victim, a possible offender profile may appear as a minor contributor. If rapid (mobile) DNA equipment will be implemented in case work, careful consideration must be given to the potential impact of missing donors in the trace results. It is recommended that alongside the RapidHit, traces should also be analyzed with a more sensitive technique in order to not lose information.

## 5. Conclusions

The duration of the investigation process in cases where the decentral rapid DNA procedure was deployed has been significantly reduced compared to cases where the regular procedure was used. Most of the delay in the regular process lies in the procedural steps during the police investigation, not in the DNA analysis. This highlights the importance of an effective work process and having sufficient capacity available. Rather than focusing on technological solutions, improved turnaround times can be achieved by dedicated innovations in operational procedures.

This study shows, in correspondence with known literature, that rapid DNA techniques are less sensitive than regular DNA analysis equipment. Comparison between the RapidHit 200 and regular DNA analysis shows that especially saliva traces secured at a crime scene should be selected critically with regard to the potential limited DNA quantity (success rate) before analyzing them with a rapid DNA technology. The rapid equipment is therefore, to a limited extent, suitable for the analysis of saliva traces secured at the crime scene and can mainly be used for the analysis of visible blood traces with an expected high DNA quantity of a single donor.

Incorporating rapid DNA analysis equipment in real casework could be promising. This study has shown that rapid results have led to multiple quick identifications of suspects, especially in volume crime cases. However, the quality of the DNA profiles generated using the RapidHit are still far from desirable compared to the results obtained by the regular procedure. Due to the lower sensitivity of the RapidHit and the inconsistent results, particularly of saliva traces, it is necessary that crime scene traces are also examined in the laboratory to prevent loss of information until more advanced equipment is available.

The acceleration in the procedure is largely dependent on an efficient work process. The question remains whether the achieved results are opportune (enough) to invest in further development of this procedure for the analysis of real crime scene traces with the current available technology. Additional research is highly recommended to evaluate other equipment and sampling methods and develop criteria for selecting crime scene traces that are suitable for the less sensitive rapid (mobile) DNA procedure. Next to this, it should be kept in mind that rapid technologies and the choice of mobile solutions are only part of the whole range of possibilities to explore the best set of methods and procedures to meet the needs for rapid and effective investigations.

## Data Availability

The data presented in this study are not available due to privacy restrictions.
